# *KMT2A* alterations in acute myeloid leukemia: a proposed genetic risk model and transplantation outcomes

**DOI:** 10.1186/s40164-025-00714-8

**Published:** 2025-10-21

**Authors:** Li Chen, Jianfeng Li, Yongmei Zhu, Xiangqin Weng, Yuting Huang, Lingling Zhao, Guang Yang, Ting Huang, Ran An, Zhiyin Liu, Xiaoqian Xu, Yubao Chen, Qiuhua Huang, Kankan Wang, Sujiang Zhang

**Affiliations:** 1 Shanghai Institute of Hematology, State Key Laboratory of Medical Genomics, National Research Center for Translational Medicine at Shanghai, Ruijin Hospital, Shanghai Jiao Tong University School of Medicine, Shanghai, China; 2 Department of Hematology, Ruijin Hospital, Shanghai Jiao Tong University School of Medicine, Shanghai, China; 3 Shanghai Jiao Tong University School of Medicine, Shanghai, China

**Keywords:** *KMT2A* rearrangement (*KMT2A*-r), *KMT2A* partial tandem duplication (*KMT2A*-PTD), Acute myeloid leukemia (AML), Allogeneic hematopoietic cell transplantation (allo-HCT), Risk stratification

## Abstract

*KMT2A*-altered acute myeloid leukemia (AML) comprises rearrangements (*KMT2A*-r), partial tandem duplications (*KMT2A*-PTD), and dual alterations (*KMT2A*-r/PTD). In this study of 125 patients, these subgroups exhibited distinct molecular profiles: *KMT2A*-r cases were enriched in RAS pathway mutations, whereas *KMT2A*-PTD showed a higher burden of epigenetic alterations. Although overall survival (OS) and event-free survival (EFS) did not differ significantly between subgroups, prognosis was strongly influenced by fusion partners. *MLLT3/ELL*-rearranged cases showed superior outcomes, but concurrent *KMT2A*-PTD abrogated this survival advantage, *AFDN* and other fusions showed poor outcomes. We therefore propose a revised three-tier risk model integrating fusion partner and PTD status, which significantly stratified patient outcomes. The intermediate-risk group (*MLLT3/ELL* without PTD) had a 3-year OS of 78.1%, compared to 50.5% in the high-risk group (all PTD), and 34.9% in the very high-risk group (other *KMT2A*-r) (*P* = 0.044). For EFS, the rates were 71.0%, 40.1%, and 24.9%, respectively (*P* = 0.003). Allogeneic hematopoietic cell transplantation significantly improved survival, with 3-year OS rates of 75.2% in transplant recipients versus 22.5% in non-transplanted patients (*P* < 0.001), particularly in high-risk groups and when performed in first complete remission. These findings support the use of molecularly guided, risk-adapted therapy in *KMT2A*-altered AML.

**Supplementary Information:**

The online version contains supplementary material available at 10.1186/s40164-025-00714-8.

**To the editor:**

Lysine methyltransferase 2A (*KMT2A*) alterations constitute a heterogeneous group in acute myeloid leukemia (AML) [1]. We characterized 125 AML patients treated at our center between February 2019 and May 2023, classifying them into *KMT2A* rearrangements (*KMT2A*-r, 36%), partial tandem duplications* (KMT2A*-PTD, 53%), or dual alterations (*KMT2A*-r/PTD, 11%), each exhibiting distinct clinical and molecular profiles (Fig. 1A).

**Fig. 1:**
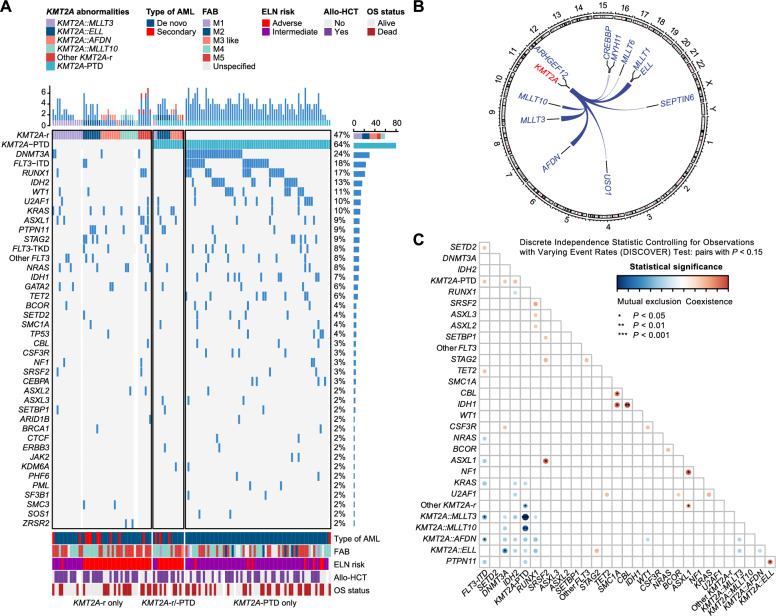
Genomic characterization in *KMT2A*-related acute myeloid leukemia (AML). **A** Genetic landscape in *KMT2A*-related AML. Each column represents a patient and each row corresponds to a gene. The color of each rectangle represents the type of gene mutation. **B** A circos plot of all *KMT2A* fusions. Chromosomes are arranged in a clockwise manner, and fusion gene pairs are connected using ribbons (for recurrent events) or lines (for single events). **C** Heatmap of mutual exclusion and co-occurrence patterns between *KMT2A* alterations and sequence variants showing representative gene pairs (*P* < 0.15). The heatmap shows the statistically significant level of co-occurrence and mutual exclusivity analysis. Blue represents mutual exclusivity, while red represents co-occurrence. The darker the color, the more significant the statistical analysis of co-occurrence and mutual exclusivity

*KMT2A*-r patients were younger and had a higher incidence of secondary AML, lower platelet counts, and higher bone marrow blast percentages than *KMT2A*-PTD patients, while *KMT2A*-r/PTD showed intermediate features (Table S1).

Genomic profiling revealed *MLLT3* (31%), *AFDN* (20%), *ELL* (18%), and *MLLT10* (18%) as major *KMT2A*-r fusions, with *KMT2A*-r/PTD enriched in *ELL* (43%) and *AFDN* (36%) (Fig. 1B, S1; Table S2). Despite intermediate clinical features, *KMT2A*-r/PTD molecularly resembled *KMT2A*-r, suggesting *KMT2A*-r initiates leukemia and *KMT2A*-PTD accelerates progression [2–4]. Methods are detailed in Supplementary Materials. *KMT2A*-r cases exhibited fewer mutations but were enriched for RAS pathway alterations (*KRAS/NRAS/PTPN11*; cumulative 47.6%) [4–6]. In contrast, *KMT2A*-PTD cases showed higher mutational burden, especially in epigenetic regulators (e.g., *DNMT3A* 40%, *FLT3*-ITD 32.3%, *RUNX1* 26.2%, *IDH2* 24.6%), consistent with clonal evolution from pre-leukemic lesions [7–9]. *KMT2A*-r/PTD retained a RAS-dominant signature (*KRAS* 21.4%, *PTPN11* 14.3%, *NRAS* 7.1%), further supporting the hierarchical model (Table S3, Fig. 1A). Co-mutated patterns are visualized in Fig. 1C and S2. We found 6 mutually exclusive and 7 co-occurring gene pairs (*P* < 0.05). *KMT2A*-PTD strongly excluded both *KMT2A::MLLT3* and *KMT2A::MLLT10*, even after statistical correction (*Q* < 0.05). By European LeukemiaNet (ELN) 2022 criteria, 59% and 41% of patients were intermediate- and adverse-risk, respectively (Table S1; Fig. 1A).

Among 120 treated patients, 77.5% received intensive and 22.5% less-intensive chemotherapy (Table S4). The composite complete remission rate was 54% after initial induction, increasing to 69% post-second induction, with no significant differences across subgroups or treatments (Table S1, S5).

With median follow-up of 16.9 months (range 0.2–54.5), the cohort demonstrated 3-year overall survival (OS) of 51.2% (median: 38.6 months) and event-free survival (EFS) of 42.0% (median: 23.7 months), respectively. Neither ELN 2022 stratification nor *KMT2A*-altered subgroups showed significant differences in survival (Fig. S3; 2A-B). However, within *KMT2A*-r, prognosis was fusion-dependent: *MLLT3*/*ELL* fusions showed superior EFS (median not reached), whereas *AFDN* or others had poorer outcomes (median: 3.9, 7.3 months). Concurrent *KMT2A*-PTD abrogated the survival advantage of *MLLT3/ELL* fusions (median: 17.6 months, *P* = 0.016) (Fig. 2C). This may reflect *KMT2A*-PTD-mediated epigenetic dysregulation of HOXA genes, overriding the favorable biology of specific fusion partners [10].

**Fig. 2:**
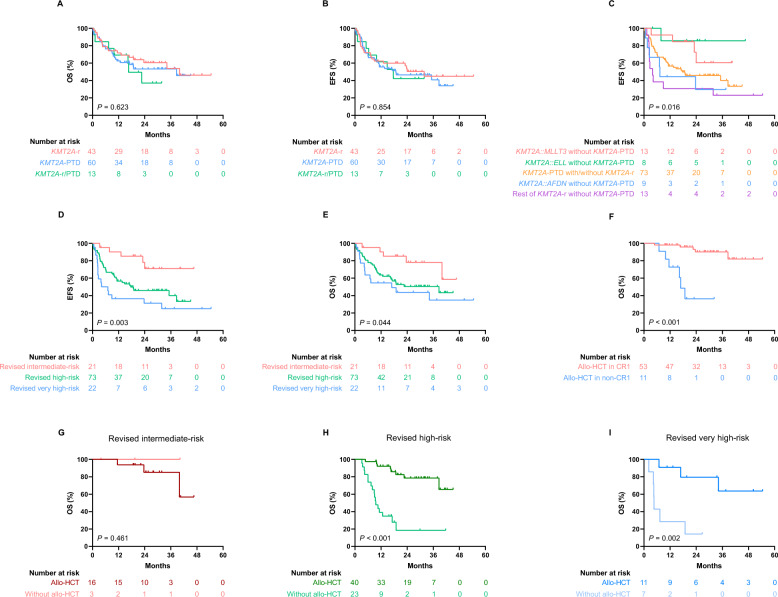
Survival and transplant outcomes in *KMT2A*-altered acute myeloid leukemia (AML). **A**–**C** Kaplan–Meier curves comparing survival across molecular subtypes. **D**, **E** Survival analysis based on the revised three-tier risk model. **F** Impact of allogeneic hematopoietic cell transplantation (allo-HCT) on overall survival in patients receiving transplant in first complete remission (CR1) versus non-CR1 status. **G**–**I** Outcomes of allo-HCT within each revised risk subgroup

We therefore propose a revised three-tier risk model that significantly stratified survival. The intermediate-risk group (*MLLT3/ELL*-rearranged without PTD) exhibited a favorable 3-year OS of 78.1% (median not reached), compared to 50.5% (median: 38.6 months) in the high-risk group (all PTD), and 34.9% (median: 16.9 months) in the very high-risk group (other *KMT2A*-r) (*P* = 0.044). For EFS, rates were 71.0% (median not reached), 40.1% (median: 17.6 months), and 24.9% (median: 4.2 months), respectively (*P* = 0.003) (Fig. 2D–E). Time-dependent receiver operating characteristics analysis confirmed superior predictive accuracy over ELN 2022, with higher area under the curve values for both OS (0.632 versus 0.590) and EFS (0.637 versus 0.547) (Fig. S4). Exploratory analyses linked *KRAS* mutations in *KMT2A*-r (n = 6) and *FLT3*-TKD in *KMT2A*-PTD (n = 7) to shorter EFS (*P* < 0.05) but not OS (Fig. S5), though validation in larger cohorts was warranted due to limited sample size.

Allogeneic hematopoietic cell transplantation (allo-HCT) significantly improved survival versus non-HCT (3-year OS: 75.2% versus 22.5%; EFS: 66.1% versus 11.5%, both *P* < 0.001). Patients receiving allo-HCT in first complete remission (CR1; 78% of HCT cases) had superior outcomes, with a 32-month OS of 90.2% versus 36.4% for non-CR1 patients (*P* < 0.001) (Fig. 2F). The benefit of allo-HCT was confined to high-risk (*P* < 0.001) and very high-risk patients (*P* = 0.002), but not intermediate-risk cases (*P* = 0.461) (Fig. 2G–I). Multivariate analysis identified achieving CR after two induction cycles and allo-HCT receipt as independent favorable predictors for OS and EFS (Table S6).

Study limitations include small sample sizes in subgroups (e.g., *KMT2A*-r/PTD) and potential biases inherent to single-center retrospective designs.

These findings advocate for risk-adapted therapy in *KMT2A*-altered AML, prioritizing early transplantation in high-risk patients and novel agents like menin inhibitors.

## Supplementary Information

Supplementary Material 1.

Supplementary Material 2.

## Data Availability

Data are available upon reasonable request from the corresponding author due to privacy/ethical restrictions.

## Abbreviations

*KMT2A*: Lysine methyltransferase 2A
*AML*: Acute myeloid leukemia
*KMT2A-r*: KMT2A rearrangements
*KMT2A-PTD*: KMT2A partial tandem duplication
sAML: Secondary AML
ELN: European Leukemia Net
OS: Overall survival
EFS: Event-free survival
Allo-HCT: Allogeneic hematopoietic cell transplantation
CR1: First complete remission
