# Supplementary material for: KMT2A alterations in acute myeloid leukemia: a proposed genetic risk model and transplantation outcomes
Source: Exp Hematol Oncol. 2025 Oct 21;14:123. doi: 10.1186/s40164-025-00714-8 (PMC12539010; doi:10.1186/s40164-025-00714-8)
Supplement: Supplementary file 1 — Supplementary Material 1. [file 40164_2025_714_MOESM1_ESM.pdf]

Supplementary information for

Title: *KMT2A* Alterations in Acute Myeloid Leukemia: A Proposed Genetic Risk Model and Transplantation Outcomes

Methods

Patient inclusion criteria and cohort definition

We conducted a retrospective analysis of newly diagnosed acute myeloid leukemia (AML) patients with *KMT2A*-r and/or *KMT2A*-PTD treated at our center from February 2019 to May 2023. All patients provided written informed consent, and the study was approved by the Ethics Committee of Ruijin Hospital in Shanghai, in accordance with the Declaration of Helsinki.

Bone marrow examination and initial diagnostic workup

All patients underwent diagnostic bone marrow (BM) evaluation at the Shanghai Institute of Hematology, including morphology assessment, flow cytometric immunophenotyping, cytogenetic analysis, and molecular profiling. Chromosome testing was performed using RHG-banding, and results were interpreted according to the International System for Human Cytogenomic Nomenclature (ISCN).

Nucleic acid extraction and sequencing library preparation

A total of 121 patients underwent RNA sequencing and targeted sequencing of 100 leukemia-associated genes for sequence variant detection (see Supplementary Table S3 for gene list). BM mononuclear cells were isolated by Ficoll density gradient centrifugation, from which genomic DNA and total RNA were extracted by using the QIAamp DNA Mini Kit (Qiagen) or TRIzol reagent (Invitrogen) according to the manufacturer's instructions. The quality and quantity of DNA/RNA were respectively evaluated by the Agilent 2100 Bioanalyzer system (Agilent Technologies) and Qubit (Life Technologies) before library preparation.

Libraries for RNA sequencing were constructed with KAPA mRNA HyperPrep Kit (Roche) and were sequenced on the NovaSeq 6000 platform (Illumina). Hybrid capture-based targeted sequencing was performed on the coding sequence of acute leukemia-related genes. For targeted DNA sequencing, 300 ng of genomic DNA per sample was sheared with Covaris E220 Focused-ultrasonicator. Libraries were firstly carried out using the NadPrep DNA Library Preparation Kit (Nanodigmbio), then the prepared gDNA libraries were hybridized with probes synthesized by IDT®, finally the capture libraries were sequenced on a NextSeq 550 platform (Illumina). Detailed protocols were implemented as previously described [1].

Bioinformatic Processing and Variant Analysis

Two methods including fusioncatcher (v1.33) and arriba (v2.4.0) [2] were used to call gene fusions from RNA-Seq data. The schematic diagram of the gene fusion protein was drawn using ProteinPaint [3].

For targeted DNA sequencing, raw paired-end reads were preprocessed with fastp

(v1.0.1) [4] and aligned to the human reference genome (hg19) using BWA (v0.7.17). Somatic variant calling was performed using an ensemble approach incorporating GATK HaplotypeCaller (v4.6.2.0), GATK UnifiedGenoTyper (v3.8.0), LoFreq (v2.1.2) [5], FreeBayes (v.1.3.2), VarDict (v1.8.3) [6], VarScan2 (v2.4.6), Strelka2 (v2.9.10), and Pindel (v0.2.5b9). The resulting variant calls were consolidated into a unified variant call format (VCF) file, which was then normalized to comply with the Human Genome Variation Society (HGVS) 3' rule conventions using BCFtools (v1.22) [7], and variants were merged based on genotypes calls and maximum variant allele frequency (VAF). The generated VCF files were annotated and converted to MAF format files by using the VEP (v114) [8] and vcf2maf (v0d3f514). For other filtering criteria, please refer to our previously published works [9,10]. Finally, the Discrete Independence Statistic Controlling for Observations with Varying Event Rates algorithm was used in rigorous statistical analysis for mutually exclusive and coexisting gene pairs [11].

Target-enrichment panel design

The custom 100-gene panel was designed to cover coding regions of genes recurrently altered in acute leukemias. Gene selection was informed by clinical guidelines from the European LeukemiaNet (ELN) [12] and National Comprehensive Cancer Network (NCCN) [13], in addition to curated literature reviews [14], and prior findings from our group [15].

Definitions and statistical analysis

Complete remission (CR), overall survival (OS), and event-free survival (EFS) were defined according to international guidelines. Composite complete remission (CRc) included CR or CR with incomplete hematologic recovery (CRi) [12].

Categorical variables were analyzed using Pearson's Chi-squared test or Fisher's exact test, while continuous variables were compared with the t-test. OS and EFS were estimated using the Kaplan–Meier method and compared by the log-rank test. Cox proportional hazards regression was performed to identify potential risk factors for survival. All analyses were two-sided with a 5% significance level. Statistical analyses were conducted using SPSS version 29.0. Time-dependent receiver operating characteristic (ROC) analysis was performed to evaluate the predictive accuracy of the model. Using R software (version 4.4.2) with the timeROC package, 3-year ROC curves for OS and EFS were plotted and the corresponding area under the curve (AUC) values were calculated. DeLong's test was applied to compare AUC differences between the newly developed model and the ELN 2022 risk stratification at the 3-year time point to statistically assess their comparative predictive performance.

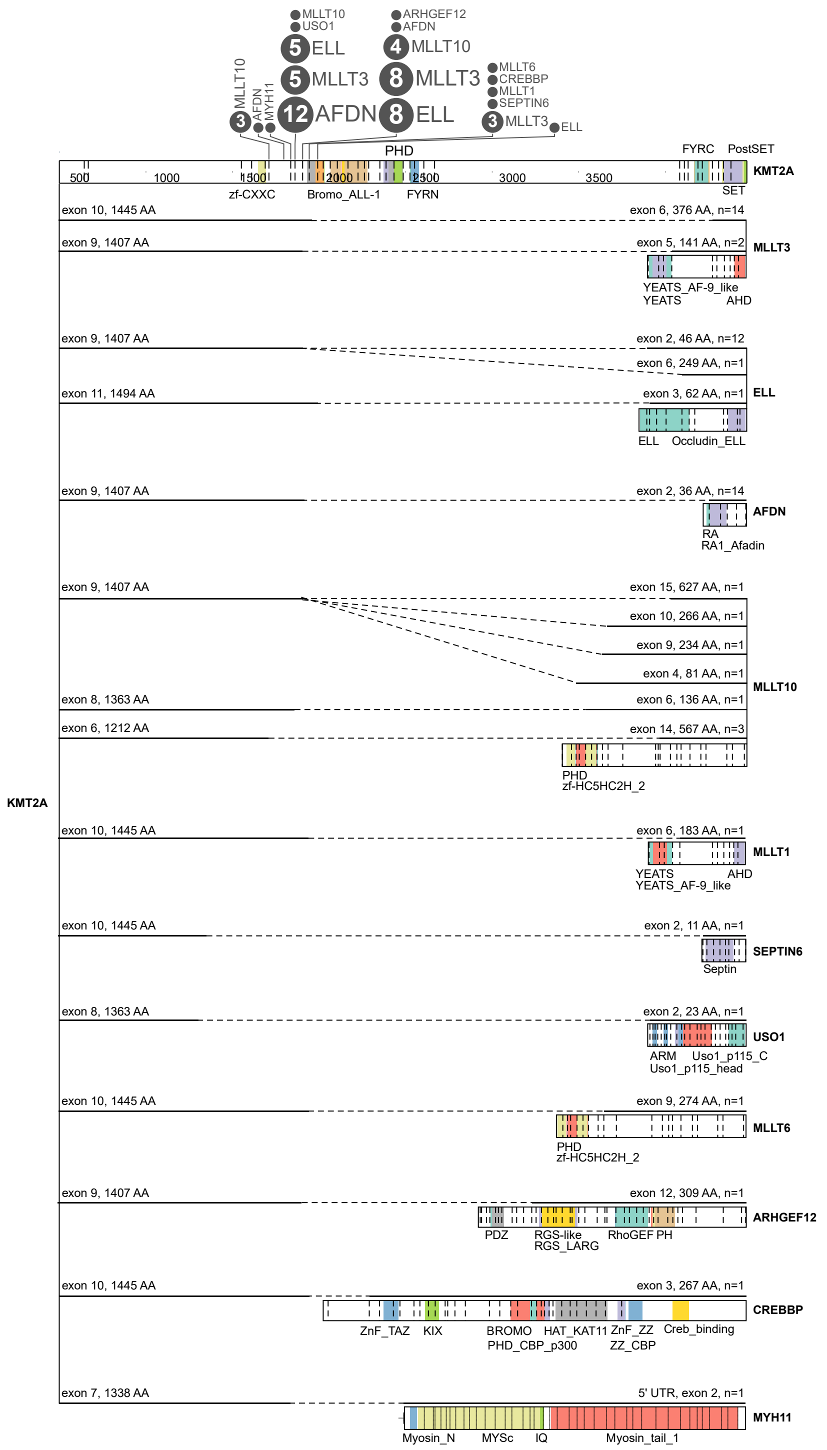

Fig. S1 Schematic diagram of major *KMT2A* gene fusion events in 59 patients with acute myeloid leukemia (AML). At the top is the full-length *KMT2A* protein. The types and quantities of fusion events are labeled with bubbles and numbers. On the left are the *KMT2A* gene breakpoints, with solid lines representing the retained segments. On the right are the protein pattern diagrams of *KMT2A* fusion partner genes and their breakpoints. Dashed lines indicate that fusion proteins are generated at these breakpoints.

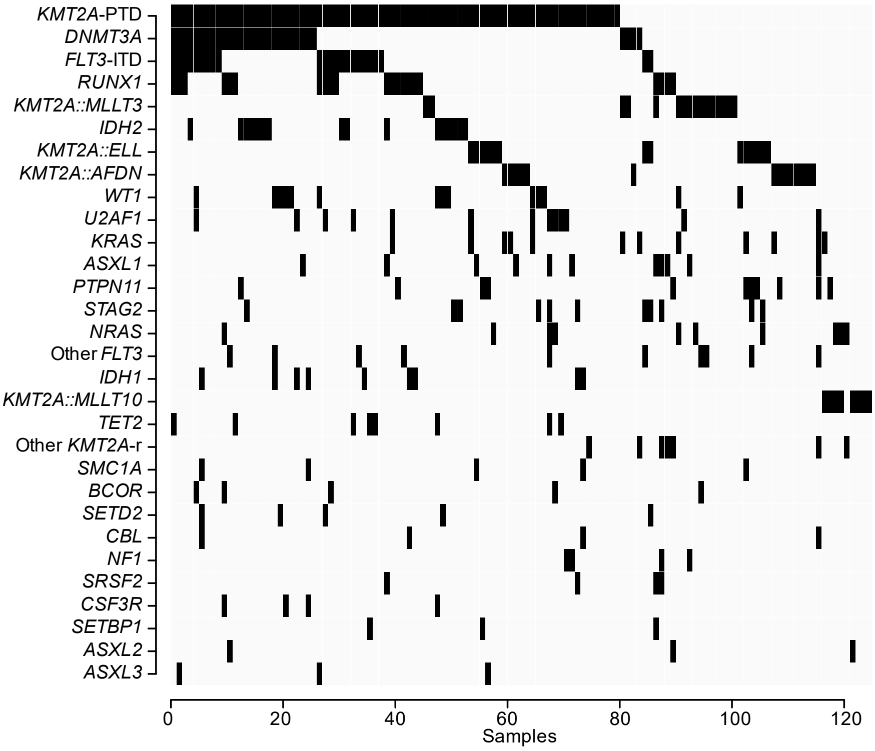

Fig. S2 Oncoplot of mutual exclusion and co-occurrence patterns between *KMT2A* alterations and sequence variants showing representative gene pairs ($P < 0.15$).

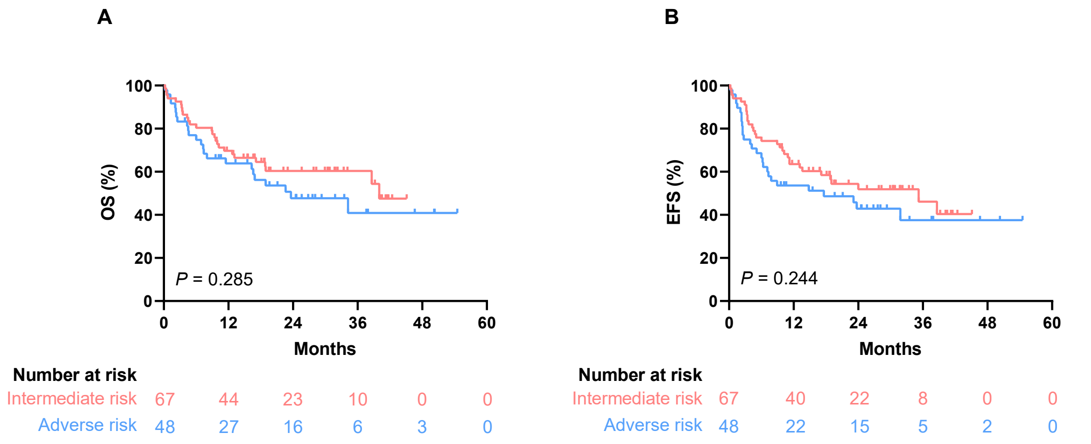

Fig. S3 Survival outcomes stratified by European LeukemiaNet (ELN) 2022 risk criteria. (A) overall survival (OS), (B) event-free survival (EFS).

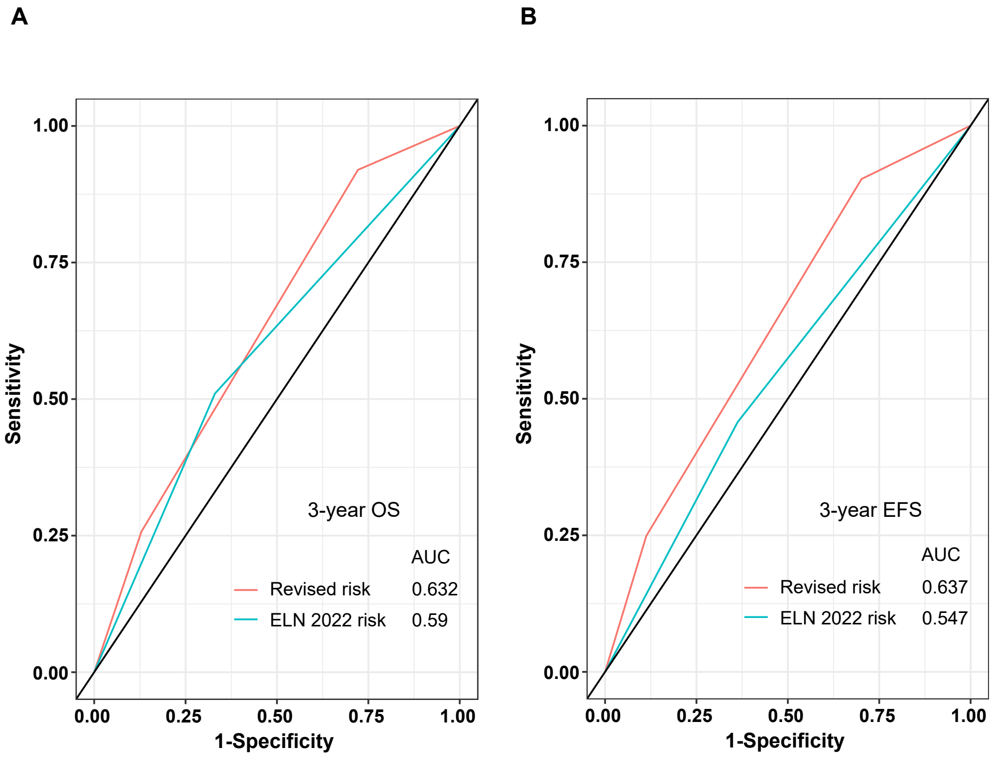

Fig. S4 Predictive accuracy comparison between the revised model and ELN 2022 criteria. (A) Time-dependent ROC curves for 3-year overall survival (OS). (B) Time-dependent ROC curves for 3-year event-free survival (EFS). ELN, European LeukemiaNet; ROC, receiver operating characteristics; AUC, area under the curve.

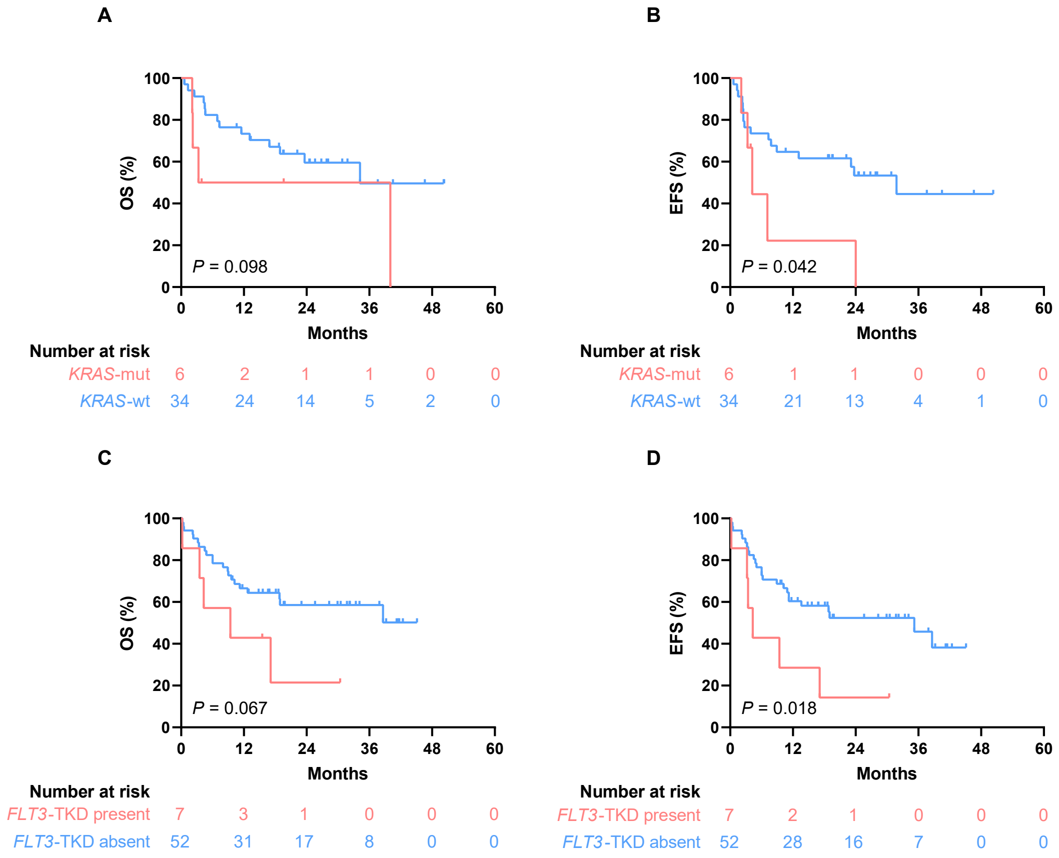

Fig. S5 Prognostic impact of co-occurring mutations in *KMT2A*-altered acute myeloid leukemia (AML). (A-B) *KRAS* mutations in *KMT2A*-rearranged AML. (C-D) *FLT3*-TKD mutations in *KMT2A*-PTD AML.

Detailed data for Tables S1 through S6 are provided in a separate Excel file.

References

1. Cheng WY, Li JF, Zhu YM, et al. Transcriptome-based molecular subtypes and differentiation hierarchies improve the classification framework of acute myeloid leukemia. *Proc Natl Acad Sci U S A* 2022; 119(49): e2211429119.
2. Uhrig S, Ellermann J, Walther T, et al. Accurate and efficient detection of gene fusions from RNA sequencing data. *Genome Res* 2021; 31(3): 448-60.
3. Zhou X, Edmonson MN, Wilkinson MR, et al. Exploring genomic alteration in pediatric cancer using ProteinPaint. *Nat Genet* 2016; 48(1): 4-6.
4. Chen S. Ultrafast one-pass FASTQ data preprocessing, quality control, and deduplication using fastp. *Imeta* 2023; 2(2): e107.
5. Wilm A, Aw PP, Bertrand D, et al. LoFreq: a sequence-quality aware, ultra-sensitive variant caller for uncovering cell-population heterogeneity from high-throughput sequencing datasets. *Nucleic Acids Res* 2012; 40(22): 11189-201.
6. Lai Z, Markovets A, Ahdesmaki M, et al. VarDict: a novel and versatile variant caller for next-generation sequencing in cancer research. *Nucleic Acids Res* 2016; 44(11): e108.
7. den Dunnen JT, Dalgleish R, Maglott DR, et al. HGVS Recommendations for the Description of Sequence Variants: 2016 Update. *Hum Mutat* 2016; 37(6): 564-9.
8. McLaren W, Gil L, Hunt SE, et al. The Ensembl Variant Effect Predictor. *Genome Biol* 2016; 17(1): 122.
9. Cheng W-Y, Li J-F, Zhu Y-M, et al. Transcriptome-based molecular subtypes and differentiation hierarchies improve the classification framework of acute myeloid leukemia. *Proceedings of the National Academy of Sciences* 2022; 119(49): e2211429119.
10. Li JF, Cheng WY, Lin XJ, et al. Aging and comprehensive molecular profiling in acute myeloid leukemia. *Proc Natl Acad Sci U S A* 2024; 121(10): e2319366121.
11. Canisius S, Martens JW, Wessels LF. A novel independence test for somatic alterations in cancer shows that biology drives mutual exclusivity but chance explains most co-occurrence. *Genome Biol* 2016; 17(1): 261.
12. Dohner H, Estey E, Grimwade D, et al. Diagnosis and management of AML in adults: 2017 ELN recommendations from an international expert panel. *Blood* 2017; 129(4): 424-47.
13. O'Donnell MR, Tallman MS, Abboud CN, et al. Acute Myeloid Leukemia, Version 3.2017, NCCN Clinical Practice Guidelines in Oncology. *J Natl Compr Canc Netw* 2017; 15(7): 926-57.
14. Papaemmanuil E, Gerstung M, Bullinger L, et al. Genomic Classification and Prognosis in Acute Myeloid Leukemia. N Engl J Med 2016; 374(23): 2209-21.
15. Yan XJ, Xu J, Gu ZH, et al. Exome sequencing identifies somatic mutations of DNA methyltransferase gene DNMT3A in acute monocytic leukemia. Nat Genet 2011; 43(4): 309-15.
